# A novel role for *CFTR* interaction with LH and *FGF* in azoospermia and epididymal maldevelopment caused by cryptorchidism

**DOI:** 10.1186/s12610-022-00160-0

**Published:** 2022-06-21

**Authors:** Faruk Hadziselimovic, Gilvydas Verkauskas, Michael Stadler

**Affiliations:** 1 Cryptorchidism Research Institute, Children’s Day Care Center Liestal, 4410 Liestal, Schweiz Switzerland; 2 Children’s Surgery Centre, Faculty of Medicine, Vilnius University, 01513 Vilnius, Lithuania; 3 Friedrich Miescher Institute for Biomedical Research, Basel, Switzerland; 4 Swiss Institute of Bioinformatics, Basel, Switzerland; 5 Faculty of Science, University of Basel, Basel, Switzerland

**Keywords:** Cryptorchidism, CFTR, Azoospermia, Epididymis, Hypogonadotropic hypogonadism, GnRHa, Cryptorchidie, CFTR, Azoospermie épididyme, GnRHa, Hypogonadisme hypogondotrope

## Abstract

Cryptorchidism occurs frequently in children with cystic fibrosis. Among boys with cryptorchidism and abrogated mini-puberty, the development of the epididymis and the vas deferens is frequently impaired. This finding suggests that a common cause underlies the abnormal development of Ad spermatogonia and the epididymis. The cystic fibrosis transmembrane conductance regulator (CFTR) is an ATP-binding cassette transporter protein that acts as a chloride channel. The *CFTR* gene has been associated with spermatogenesis and male fertility. In boys with cryptorchidism, prepubertal hypogonadotropic hypogonadism induces suboptimal expression of the ankyrin-like protein gene, *ASZ1*, the P-element induced wimpy testis-like gene, *PIWIL,* and *CFTR*. The abrogated expression of these gene leads to transposon reactivation, and ultimately, infertility. Curative gonadotropin-releasing hormone agonist (GnRHa) treatment stimulates the expression of *CFTR* and *PIWIL3*, which play important roles in the development of Ad spermatogonia and fertility. Furthermore, GnRHa stimulates the expression of the epididymal androgen-sensitive genes, *CRISP1, WFDC8, SPINK13*, and *PAX2*, which thereby promotes epididymal development. This review focuses on molecular evidence that favors a role for *CFTR* in cryptorchidism-induced infertility. Based on information available in the literature, we interpreted our RNA-Seq expression data obtained from samples before and after randomized GnRHa treatment in boys with bilateral cryptorchidism. We propose that, in boys with cryptorchidism, *CFTR* expression is controlled by luteinizing hormone and testosterone. Moreover, CFTR regulates the activities of genes that are important for fertility and Wolffian duct differentiation.

## Résumé

La cryptorchidie survient fréquemment chez les enfants atteints de mucoviscidose et une altération du développement de l’épididyme et du canal déférent a été observée chez les garçons cryptorchides avec une mini-puberté abrogée. Cela suggère que le développement anormal des spermatogonies Ad et de l’épididyme ont une cause commune. CFTR est. une protéine de transport de cassette de liaison à l’ATP qui agit comme un canal chlorure. Plus précisément, le *CFTR *a été associé à la spermatogenèse et à la fertilité masculine. Chez les garçons cryptorchides, l’hypogonadisme hypogonadotrope prépubère induit une expression sous-optimale d’*ASZ1*, de quatre gènes *PIWIL* et de *CFTR*, entraînant la réactivation des transposons et l’infertilité. Le traitement curatif à la GnRHa stimule l’expression des gènes *CFTR* et *PIWIL3* qui sont importants pour le développement des spermatogonies Ad et la fertilité subséquente. En outre. Le traitement à la GnRHa stimule les gènes épididymaires sensibles aux androgènes *CRISP1, WFDC8, SPINK13, PAX2* favorisant le développement de l’épididyme. Cette revue se concentre sur les preuves moléculaires en faveur du rôle du CFTR dans l’infertilité causée par la cryptorchidie. Nous avons interprété les données d’expression de RNAseq obtenues avec des échantillons avant et après un traitement randomisé à la GnRHa chez des garçons cryptorchides bilatéraux dans le contexte des informations disponibles dans la littérature. Nous proposons que chez les garçons cryptorchides, le *CFTR* est. contrôlé par l’hormone lutéinisante (LH) et la testostérone et influence les activités des gènes qui sont importants pour la fertilité et la différenciation du canal de Wolff.

## Introduction

**Fig. 1:**
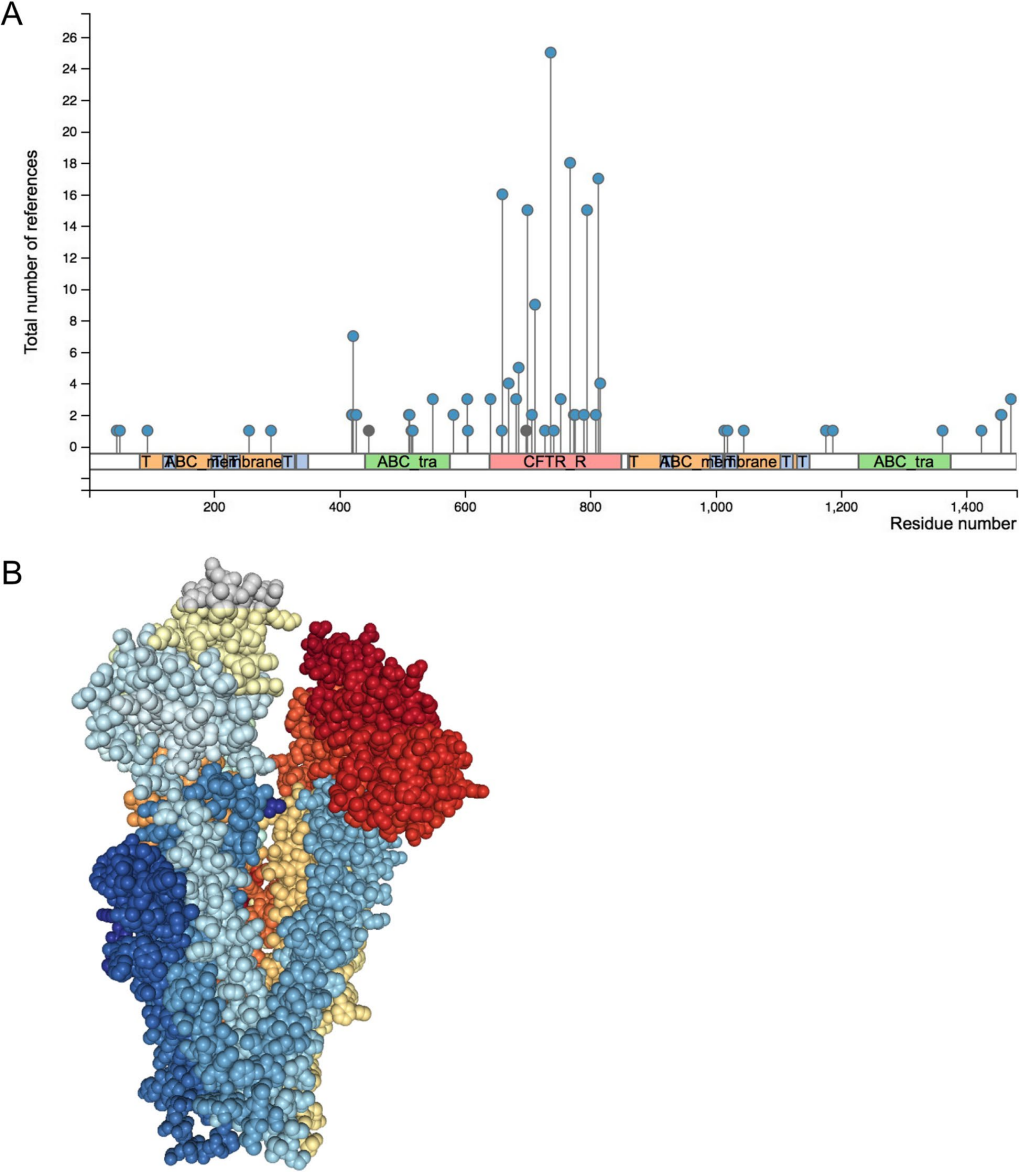
CFTR post-translational modifications and protein structure. **A** A lollipop plot (from www.phosphosite.org) shows the primary sequence, including functional domains (y-axis), plotted against the number of observations of post-translational modifications (x-axis) described in the literature. Phosphorylation is shown in blue, SUMOylation and tri-methylation are shown in green. **B** The 3D structure of CFTR, from www.rcsb.org (5UAK), is reproduced with permission from Liu et al. [1]

The cystic fibrosis transmembrane conductance regulator (CFTR) belongs to the family of ATP-binding cassette (ABC) transporter proteins, and it functions as a chloride channel. CFTR undergoes various post-translational modifications, including phosphorylation, SUMOylation, and mono-methylation (Fig. 1A; from www.phosphosite.org). Studies on the structure of CFTR revealed a helix-loop transition in transmembrane helix 8, which is likely critical for the protein’s channel function. This structure constitutes a unique feature not found in other ABC transporters (Fig. 1B) [1]. CFTR is critical for the secretion of ions and the transport of water molecules in epithelial tissues. Point mutations in the *CTFR* gene have been shown to cause cystic fibrosis. Among the best-studied mutations causes the deletion of a phenylalanine at position 508, which interferes with the folding and localization of the mutant protein [2].

CFTR is also important for fetal development, epithelial differentiation and regeneration, and the regulation of the epithelial-to-mesenchymal transition [3]. In addition, CFTR is involved in the excitability of neurons, the proper functioning of skeletal-, cardiac-, and smooth muscles, the regulation of cell volume, the transepithelial transport of salt ions, and the acidification of intra- and extracellular compartments [3].

**Fig. 2:**
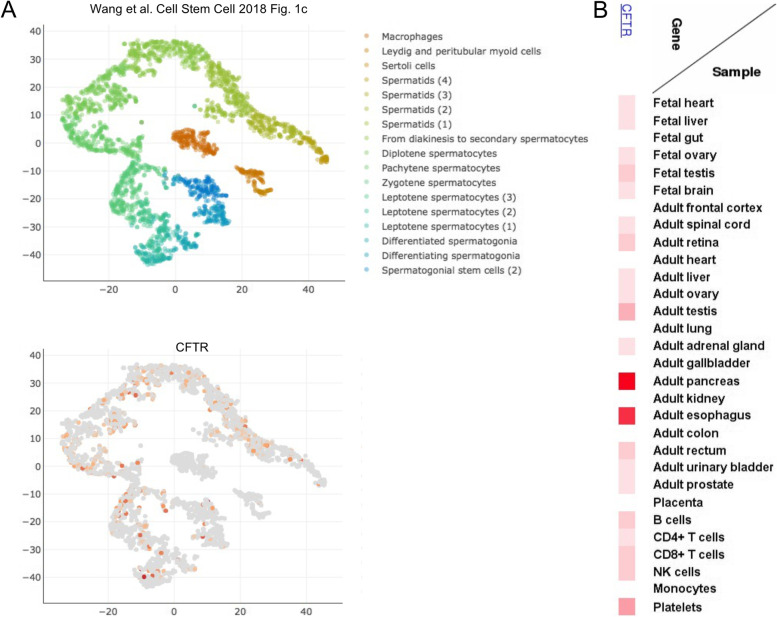
CFTR mRNA and protein expression. **A** Single-cell RNA-Seq data are from Wang et al. [13]. The images were retrieved from the Reproductive Genomics Viewer (RGV; https://rgv.genouest.org). **B** Heat map shows protein profiling data for the indicated samples; data were reported by Kim et al. [14]. The image was retrieved from www.humanproteomemap.org. The color-scale indicates the protein levels

It has been suggested that CFTR plays a role in spermiogenesis [4–6]. During this process, spermatids differentiate into spermatozoa by undergoing extensive remodeling, including chromatin condensation, acrosome formation, elongation, cell volume reduction, and flagellum formation [4]. Genetic data are in keeping with single-cell RNA sequencing (scRNA-Seq) expression data that have revealed that a sub-population of male germ cells, at different developmental stages, expressing the *CTFR* gene above the threshold level of detection (Fig. 2A). These expression data were confirmed at the protein level by mass spectrometry-based proteome profiling and immunohistochemical analysis of testicular sections (Fig. 2B) [5]. Furthermore, CFTR is also expressed in the human epididymis [6]. Human genetic studies have shown that point mutations in the *CFTR* gene were associated with non-obstructive azoospermia and impaired spermatogenesis [7, 8]. Indeed, males with cystic fibrosis caused by mutations in *CFTR* have exhibited a wide range of testicular histology, from normal to severely pathological [9, 10]. It has been suggested that defects in CFTR may result in insufficient activation of follicle stimulating hormone (FSH)-induced signal transduction and gene expression, which could lead to impaired spermatogenesis [11]. Infertile, but otherwise healthy males have *CFTR* mutations at significantly higher frequencies than the expected frequencies in the general population [12]. This observation implies that, although certain *CFTR* mutations give rise to clinical cystic fibrosis, with debilitating lung and pancreatic problems and congenital bilateral absence of the vas deferens, other *CFTR* mutations might occur in healthy men, whose only known clinical condition is reduced sperm quality [12].

A literature review of the PubMed database was performed using CFTR and cryptorchidism as query terms alone and in combination. Additional publications were identified via the reference lists in the articles found in the PubMed search. Patients, the biopsy samples, histological analyses, and the RNA-Sequencing protocol were described in detail in previous work [15–17]. The aim of this review article is to highlight the potential causative role of CTFR in adult male infertility and abnormal Wolffian duct development*.*

### Molecular processes underlying azoospermia induced by cryptorchidism

The pathogenesis of azoospermia in males with mutated *CFTR* genes might be explained by a mechanism that involves the cAMP-response element binding protein (CREB) pathway [11]. CREB protein levels are reduced in human azoospermia testes, which is consistent with the protein’s down-regulation in cystic fibrosis mouse models and in CFTR-inhibited cultured Sertoli cells [11]. In contrast, among patients with cryptorchidism and abrogated mini-puberty, who are at high risk of developing azoospermia, we observed a weak increase in *CREB1* and *ATF2 (CREB2)* expression levels, compared to the low infertility risk group (LIR), and the *CREB3* and *CREB5* expression levels remained unaltered (Table 1). Moreover, all *CREB* genes were downregulated after a curative GnRHa treatment (Table 1). Thus, in cryptorchidism-induced azoospermia, the CREB pathway appears to be less important than it is in patients diagnosed with nonobstructive azoospermia due to conditions unrelated to undescended testes [11]. CFTR is a major hub protein that physically interacts with 831 proteins (see https://thebiogrid.org), including protein involved in targeted proteolysis, epigenetics, and temperature stress response. It was claimed that CFTR was a temperature-sensitive protein. Low temperature favored the proper folding and maturation of CFTR and promoted its insertion into the cell membrane. Conversely, elevated temperatures inhibited these processes [18]. Consequently, it was proposed that the elevated testicular temperature in cryptorchidism might cause a spermatogenic defect by impairing CFTR function, which in turn, might up-regulate the COX20 pathway and disrupt the blood-testis barrier [19]. Moreover, a key part of the heat shock response is the strong upregulation of heat shock proteins (HSPs), which primarily depends on heat shock transcription factors (HSFs). Importantly, HSF genes, endoplasmic reticulum stress genes, and six out of seven HSP genes showed no increase in expression when exposed to elevated body temperatures [20] (Table 2). Furthermore, in contrast to the adult testis, prepubertal cryptorchid testes lack increases in COX20 expression before and after treatment [log2–0.67;FDR0.005], which argues against a major effect of temperature stress.

**Table 1:** *CREB*-gene family expression in LIR and HIR boys with cryptorchidism, before and after GnRHa treatment [17, 21, 22]

Gene	HIR/LIR (RPKM)	log2FC / FDR	−/+ GnRHa treatment (RPKM)	log2FC / FDR
***CREB1***	13.8/13.0	0.19/0.04	12.58/10.78	−0.68/0.004
***ATF2(CREB2)***	25.09/23.07	0.21/0.04	22.44/15.42	−0.9/0.0003
***CREB3***	11.8/13.2	n.s.	12.4/11.6	−0.60/0.008
***CREB5***	4.50/5.08	n.s.	5.40/5.16	−0.60/0.007

*LIR* low infertility risk, *HIR* high infertility risk, *FDR* false discovery rate, *RPKM* median expression values expressed in reads per kilobase per million

**Table 2:** Genes involved in PIWIL biogenesis. FDR; false discovery rate, RPKM; median expression values expressed in reads per kilobase per million [15, 23, 24]

Gene	HIR/LIR (RPKM)	log2FC / FDR	−/+ GnRHa treatment (RPKM)	log2FC / FDR
***FOXA1***	0.11/0.47	−1.59/0.006	0.19/0.71	1.15/0.03
***ASZ1***	0.75/309	−2.32/0.001	n.s.	n.s.
***HSP90AA1***	71.2/102.3	−0.53/0.01	75.6/78.1	−0.65/0.01
***HENMT1***	7.52/20.11	−1.34/0.0001	9.69/10.5	−0.7/0.004
***FKBP6***	0.47/2.16	−2.34/0.0001	0.68/1.42	n.s.
***PIWIL1***	0.39/2.21	−2.97/0.0001	n.s.	n.s.
***PIWIL2***	3.22/16.66	−1.75/0.0008	n.s.	n.s.
***PIWIL3***	0.22/0.41	−1.0/0.015	0.17/0.95	1.52/0.002
***PIWIL4***	1.72/7.32	−2.09/0.0001	n.s.	n.s.
***PNLDC1***	0.52/3.05	−2.41/0.0006	n.s.	n.s.

It was reported that Finish, but not Danish, 3-month-old boys with cryptorchidism had elevated luteinizing hormone (LH) levels, compared to healthy controls [25]. The divergent results were most likely the consequence of diagnostic failure because cryptorchidism was not confirmed with histological analyses of testicular biopsies. Nevertheless, the observed high LH levels were thought to compensate for mild Leydig cell dysfunction, which suggested that cryptorchidism resulted from primary testicular failure [26]. This statement contrasted with at least 10 different reports on LH-RH stimulation tests that demonstrated abnormally low LH responses in boys with cryptorchidism (for references see [27]). Thus, the cause of the low testosterone response is induced at the level of the hypothalamus, and the result is insufficient Leydig cell stimulation. Therefore, most published studies do not support the hypothesis, proposed by Toppari et al., which postulates that that mild Leydig cell dysfunction results from end-organ failure in cryptorchidism [26].

The foundation of the hypogonadotropic hypogonadism hypothesis was laid out in 1976, in Stresa (Italy), when we reported that hormonal values must be analyzed in the context of the presence or absence of Ad spermatogonia [28]. Thus, we grouped patients with cryptorchidism into two categories [28]. The first group comprised patients at high infertility risk (HIR), with testes that lacked Ad spermatogonia (indicating abnormal mini-puberty) and showed pathologically low LH levels, basal and upon stimulation. The other group comprised patients at low infertility risk (LIR), with testes that contained Ad spermatogonia and displayed normal plasma LH values [28]. Ad spermatogonia have a characteristic nuclear feature that distinguishes them from the other germ cells (e.g., fetal, transient, and pale-type (Ap) spermatogonia) of developmental stages [21]. This is a major transformation of gonocytes into Ad spermatogonia and is not simply another step in a succession it represents the switch from a fetal reservoir of stem cells (gonocytes) to an adult reservoir of stem cells (Ad spermatogonia), from which all future germ cells are generated [21]. Insufficient testosterone levels fail to direct gonocytes into the differentiation process in boys with defective mini-puberty, resulting in both abrogated Ad spermatogonia development and infertility [21].

To improve our understanding of cryptorchidism-induced azoospermia, we interpreted testicular GeneChip and RNA-Seq expression data on genes involved in regulating *CFTR* expression [15, 17, 21, 22, 29]. One of these regulators, *FOXA1*, facilitates transcription-factor binding to chromatin; for example, it facilitates the binding of androgen and estrogen receptors to chromatin [30]. *FOXA1* expression was reduced in samples from an HIR group, and expression was upregulated with a curative GnRHa treatment. Thus, *FOXA1* could stimulate *CFTR* expression in response to GnRHa treatment (Table 2). Another regulator of *CFTR*, *ASZ1*, plays a central role during spermatogenesis by repressing transposable elements, which is essential for genome integrity in the germline [31]. *ASZ1* is evolutionarily conserved, and it is expressed in germ cells. The ASZ1 protein acts by metabolizing Piwi-interacting RNA (piRNA). piRNA mediates the repression of transposable elements during meiosis by forming complexes composed of piRNAs and PIWI proteins; these complexes govern the methylation and subsequent repression of transposons. *ASZ1* expression is down-regulated in patients with HIR (Table 3). PIWIL biogenesis is regulated by ASZ1, HENMT*1*, *FKBP6*, and *HSP90AA1*. These genes were all down-regulated in HIR samples (Table 2). *FKBP6* acts as a co-chaperone and represses transposable elements via its interaction with *HSP90AA1*. piRNA processing is also critically dependent on poly *A*^+^-specific RNase-like domain containing 1 (PNLDC1). Men with dysfunctional *PNLDC1* and nonobstructive azoospermia showed a concomitant loss of *PIWIL1* expression [32]. While *PNLDC1* expression in HIR testes is downregulated, no increase after GnRHa treatment was observed, which is consistent with the notion that this gene is not directly involved in the development of azoospermia in cryptorchid patients (Table 2).

**Table 3:** *CFTR* RNA sequencing and GeneChip (Affymetrix) data analysis and *SLC9A*-family RNA sequencing data. FC; the log-fold changes, FDR; false discovery rate, RPKM; median expression values expressed in reads per kilobase per million, before and after GnRHa treatment [15, 21, 22]

Gene	HIR/LIR (RPKM)	log2FC / FDR	−/+ GnRHa treatment (RPKM)	log2FC / FDR	Affymetrix probeset identifier (highest variance probe set per gene)	Descended / undescended (HIR + LIR, median log2 signal)	log2FC / FDR
***CFTR***	0.24/0.79	−1.50/0.001	0.29/1.54	1.35/0.002	205043_at	3.39/3.50	n.s.
***SLC9A3R1***	0.69/2.07	−1.43/5.77E-05	n.s.	n.s.			
***SLC9A2***	n.s	n.s.	0.13/1.00	2.18/1.69E-05			
***SLC9A4***	n.s	n.s.	0.13/0.42	2.12/0.0001			
***SLC9A9-AS1***	n.s	n.s.	0.32/2.39	2.77/0.001			

Previously, we provided evidence that supported the notion that infertility in cryptorchidism is a consequence of hypogonadotropic hypogonadism-induced alterations in the PIWIL-pathway that undermine transposon repression [23, 24]. It was also shown that testosterone altered testicular function by regulating the expression of Piwi-interacting RNAs [33]. Mutant mice with insufficient testosterone secretion expressed lower levels of Miwi [34]. Moreover, mice deficient in each of the genes essential for silencing the L1 retrotransposons were sterile [35]. We found that individuals with HIR showed little or no expression of the PIWIL4 protein or seven out of the 12 *TDRD* genes that are important for spermatogenesis [23, 24]. This deficiency was accompanied by low expression of the RNA-helicases, *DDX4* and *DDX25*, which are dependent on gonadotropin and testosterone stimulation [23, 24].

Abnormal gametogenesis results from disturbed PIWIL biogenesis (which involves four *PIWIL* genes) and insufficient *ASZ1*, *FOXA1*, and *CFTR* functions (Table 2). Importantly, curative GnRHa treatment stimulates the expression of several genes involved in pituitary development and differentiation, neuronal development, and testosterone synthesis pathways. GnRHa treatment also rescues fertility by increasing the expression of *CFTR, DMRTC2, PAX7*, *BRACHYURY/T*, *TERT*, and *PIWIL3.*

### CFTR in abnormal development of the epididymis and vas deferens in cryptorchidism

It was reported that among children with cystic fibrosis the incidence of undescended testis is five to 12 times more common than that observed in a control population [36]. Furthermore, Fedder et al. found the CFTR intron variant IVS8-5 T to be associated with cryptorchidism requiring orchidopexy. However the patient cohort was not classified into HIR and LIR groups [37], Importantly, defective development of the epididymis and the vas deferens occurs more often in boys with cryptorchidism and abrogated mini-puberty, which suggests that testicular and epididymal pathologies share a common cause [38].

Mutations in *CFTR* are thought to be responsible for the absence of the vas deferens and the distal half of the epididymis. It was suggested that these structural anomalies were caused by abnormal fluid transport in the Wolffian duct, which would then fail to differentiate into the epididymis and vas deferens during post-natal stages of development [12, 39, 40]. In mice, impaired expression of *Slc9a3* (a Na/H exchanger), reduces the levels of CFTR and causes obstructive azoospermia [41]. Among patients in the HIR group, expression of SLC9A3’s regulatory cofactor *SLC9A3R1* is downregulated (Table 3). Given that GnRHa treatment upregulates SLC9A2, SLC9A4, SLC9AS1, and CFTR and that Slc9a3 mutant mice develop epididymal obstruction, we propose that these genes contribute to the development of the epididymis (Table 3).

Other genes that are important for epididymal development, such as *SCNN1A* and *SCNN1G*, are also downregulated in patients with HIR patients (Table 4). *SCNN1A* encodes a sodium channel that enables cells to generate and transmit electrical signals [42]. An increasing number of studies show that CFTR plays a role in fundamental cellular processes such as fetal development, epithelial differentiation/polarization and regeneration, and the epithelial–mesenchymal transition [43].

**Table 4:** Androgen-responsive epididymis-related genes. FDR; false discovery rate, RPKM; median expression values expressed in reads per kilobase per million [15, 21, 22]

Gene	HIR/LIR (RPKM)	log2FC / FDR	−/+ GnRHa treatment (RPKM)	log2FC / FDR
***SCNN1A***	n.s.	n.s.	0.19/0.69	1.13/0.01
***SCNN1G***	n.d.	n.d.	0.05/0.33	2.14/0.004
***CRISP1***	n.d.	n.d.	0.17/1.46	2.76/3.06E-05
***WFDC8***	n.d.	n.d.	0.18/1.28	1.98/0.003
***SPINK13***	n.d.	n.d.	0.30/1.28	1.89/0.002
***PAX2***	n.d.	n.d.	0.02/0.40	2.09/0.004
***TRPM8***	n.s.	n.s.	0.15/0.85	2.02/1.47E-05

Androgens are the primary regulators of epididymal development and function. However, a large body of evidence has suggested that growth factors also play important roles in these processes. Among others, fibroblast growth factor (FGF) is involved in the development and normal functioning of male reproductive organs, including the testis and epididymis [44]. In 2010, we reported that boys with unilateral cryptorchidism showed impaired *FGFR1* expression in the undescended testis [45]. Moreover, reduced FGFR1 protein levels were observed in cryptorchid epididymis samples from both humans and rodents [46]. These findings suggested that FGFR1 regulates the development of the epididymal mesenchyme. It appears likely that, in humans and rodents with cryptorchidism, the impairment in FGFR1 protein secretion in the abnormal mesenchyme contributes to epididymis malformation and the lack of epididymis-testes descent. Interestingly, *FGFR* signaling regulates specific chaperones that control CFTR maturation [39].

LH is necessary for epididymis-testicular descent. LH-receptor knockout mice exhibit bilateral cryptorchidism that can be corrected with testosterone replacement therapy. Specifically, this therapy reverses morphological alterations and changes in gene expression in the knockout mice, except those related to insulin-like factor 3. This finding suggests that testosterone, rather than INSL3, facilitates the completion of testicular descent [47]. Furthermore, in 66% of naturally cryptorchid mice, treatment with LH-releasing hormone induces epididymis-testicular descent, increases testosterone secretion, and normalizes the altered morphology of the cryptorchid epididymis [48]. In boys with cryptorchidism, GnRHa treatment increases testosterone secretion, stimulates epididymis development, and induces completion of the epididymis-testicular descent [49]. Most patients who fail to respond to hormonal treatment have small, irregular epididymides [49].

**Fig. 3:**
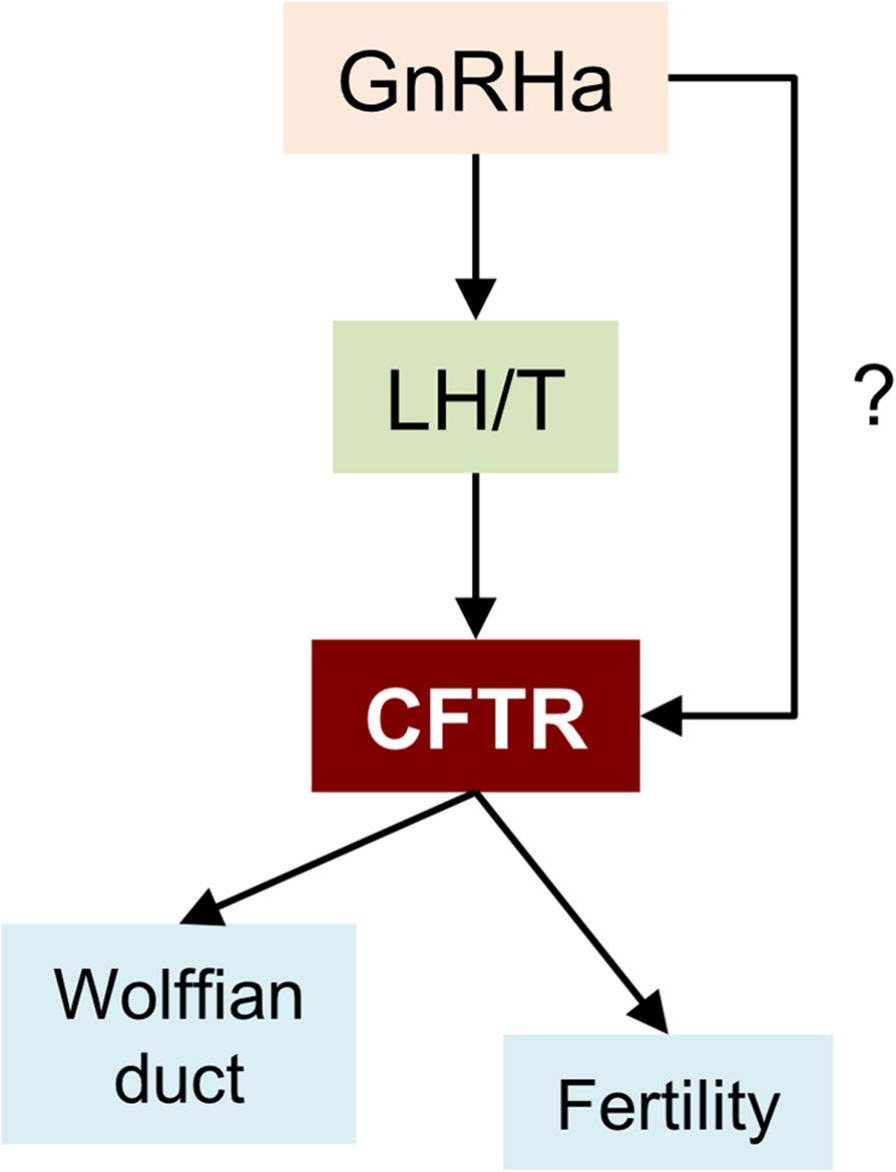
A model of CFTR and cryptorchidism-induced infertility. A schematic diagram outlines the relationships between CFTR and biological processes. A disturbance in any of the steps results in infertility, epididymal maldevelopment, and cryptorchidism

The presence of GnRH and GnRH receptor mRNAs in normal human non-reproductive tissues suggests that, in addition to regulating gonadotropin secretion from the anterior pituitary, these proteins play an important role in regulating cellular functions in an autocrine or paracrine manner [50]. The hypogonadotropic manifestations of cystic fibrosis may be partly explained by abnormal neuropeptide-vesicle trafficking, due to *CFTR* mutations [51]. Indeed, a six-month treatment with GnRHa normalized both pituitary function and *CFTR* expression. In addition, GnRHa treatment promoted chloride-channel function in F508del-CFTR cells, by increasing the stability of CFTR in the membrane [52]. Thus, it is possible that GnRHa directly regulates GnRH-dependent chloride transport in cystic fibrosis (Fig. 3). Consequently, it was suggested that both topical and intra-nasal applications of GnRHa may be potentially beneficial for treating cystic fibrosis [53].

GnRHa treatment also stimulates LH release, which in turn, stimulates testosterone release. Testosterone stimulates the expression of *CFTR* (Fig. 3) and other androgen-sensitive epidydimal genes, such as *CRISP1*, *WFDC8*, *SPINK13*, *PAX2* (Table 4), and the epithelial sodium channel subunits, *SCNN1A* and *SCNN1G* (Table 4). Thus, GnRHa therapy contributes to rescuing fertility and improving the morphology and function of the epididymis through numerous pathways (Fig. 3).

In conclusion, the high incidence of abnormal epididymides in patients with HIR may stem from a combination of a degree of prepubertal hypogonadotropic hypogonadism, insufficient CFTR activity, and FGFR1 deficiency. In addition, given that the bronchial system expresses GnRH-R [53], Buserelin could potentially be useful for treating cystic fibrosis in general and for treating boys with cryptorchidism combined with cystic fibrosis, in particular.

## Data Availability

Not applicable.

## Abbreviations

*ASZ1*: Ankyrin Repeat SAM And Basic Leucine Zipper Domain Containing 1
*BRACHYURY*: T-Box Transcription Factor T
*CFTR*: Cystic Fibrosis Transmembrane Conductance Regulator
*COX2*: Cytochrome C Oxidase Assembly Factor COX20
*CREB1*: cAMP Responsive Element Binding Protein 1
*CREB2(ATF2*): cAMP Responsive Element Binding Protein 2
*CREB3*: cAMP Responsive Element Binding Protein 3
*CREB5*: cAMP Responsive Element Binding Protein 5
*CRISP1*: Cysteine Rich Secretory Protein 1
*CTTNBP2*: Cortactin Binding Protein 2
*DDX4/25*: DEAD-Box Helicase 4/25
*DMRTC2*: DMRT-Like Family C2
*FGFR1*: Fibroblast Growth Factor Receptor 1
*FKBP6 KBP*: Prolyl Isomerase Family Member 6 (Inactive)
*FOXA1*: Fork-head Box A1
GnRHa: Gonadotropin releasing hormone agonist
*HENMT1*: HEN Methyltransferase 1
HIR: High infertility risk group
*HSF*: Heat Shock Transcription Factor
HSP90AA1: Heat Shock Transcription Factor 1
*INSL3*: Insulin-Like 3
LIR: Low infertility risk group
LH: Luteinizing hormone
*PAX2/7*: Paired Box 2/7
*PIWIL* 1–4: Piwi-Like RNA-Mediated Gene Silencing 1–4
*PNLDC1*: PARN-Like Ribonuclease Domain Containing Exonuclease 1
*SCNN1A*: Sodium Channel Epithelial 1 Subunit Alpha
*SCNN1G*: Sodium Channel Epithelial 1 Subunit Gamma
*SLC9A3*: Solute Carrier Family 9 Member A3
*SPINK13*: Serine Peptidase Inhibitor Kazal Type 13
*TERT*: Telomerase Reverse Transcriptase
*WFDC8*: WAP Four-Disulfide Core Domain 8
